# Collectanea Medica

**Published:** 1812-11

**Authors:** 


### COLLECTANEA MEDICA,

CONSISTING OF

ANECDOTES, FACTS, EXTRACTS, ILLUSTRATIONS, QUERIES, SUGGESTIONS, &c.

RELATING TO THE

*History or the Art of Medicine, and the Auxiliary Sciences.*

---

*Chemical Researches on the Blood, and some other Animal Fluids. By* WILLIAM THOMAS BRANDE, Esq. F.R.S. *Communicated to the Society for the Improvement of Animal Chemistry, and by them to the Royal Society.*

Section I.—*Introduction.*

IN the following pages, I shall have the honor of laying before this Society an account of some experiments upon the blood, which were originally undertaken with a view to ascertain the nature of its coloring matter. The difficulties attendant on the analysis of animal substances, have rendered some of the results less decisive than I could have wished; but I trust that the general conclusions to which they lead, will will be deemed of sufficient importance to occupy the time of this body.

The existence of iron in the blood was first noticed by Menghini*, and its peculiar red color has been more recently attributed to a combination of that metal with phosphoric acid, by MM. Fourcroy and Vauquelin.† The very slight discoloration occasioned by the addition of infusion of galls to a solution of the coloring matter, under circumstances most favorable to the action of that delicate test of iron, first led me to doubt the inferences of those able chemists; and subsequent experiments upon the combinations to which they allude, tended to confirm my suspicion, and induced me to give up no inconsiderable portion of the time which has elapsed since the last meeting of this Society, to the present investigation.

An examination of the chyle and of lymph, in order to compare their composition with that of the blood, formed an important part of this inquiry, especially as those fluids have not hitherto been submitted to any accurate analysis, on account of the difficulty of procuring them in sufficient quantities, and in a state of purity. Whilst engaged in assisting Mr. Home in his physiological researches, several opportunities occurred of collecting the contents of the thoracic duct under various circumstances, and in different animals; on other occasions Mr. Brodie has kindly furnished me with the materials for experiment.

### Section II.—*On the Composition of Chyle.*

The contents of the thoracic duct are subject to much variation. About four hours after an animal has taken food, provided digestion has not been interrupted, the fluid in the duct may be regarded as pure chyle; it is seen entering by the lacteals in considerable abundance, and is of an uniform whiteness throughout. At longer periods after a meal, the quantity of chyle begins to diminish, the appearance of the fluid in the duct is similar to that of milk and water; and lastly, where the animal has fasted for twenty-four hours or longer, the thoracic duct contains a transparent fluid which is pure lymph.

A. The chyle has the following properties:

1. When collected without any admixture of blood, it is an opake fluid of a perfectly white color, without smell,

---

* Vincentius Menghinus de Ferrearum Particularum Progressu in Sanguinem. *Comment. Acad. Bonon.* t. ii. p. 2, pag. 475.

† *Système des Conn. Chym.* vol. viii.

 and and having a slightly salt taste, accompanied by a degree of sweetness.

2. The color of litmus is not affected by it, nor that of paper stained with turmeric, but it slowly changes the blue color of infusion of violets to green.

3. Its specific gravity is somewhat greater than that of water, but less than that of blood; this, however, is probably liable to much variation.

4. In about ten minutes after it is removed from the duct, it assumes the appearance of a stiff jelly, which in the course of twenty-four hours gradually separates into two parts, producing a firm and contracted coagulum, surrounded by a transparent colorless fluid. These spontaneous changes, which I have observed in every instance where the chyle was examined at a proper period after taking food, are very similar to the coagulation of the blood and its subsequent separation into serum and crassamentum; they are also retarded and accelerated by similar means.

B. 1. The coagulated portion bears a nearer resemblance to the caseous part of milk, than to the fibrine of the blood.

2. It is rapidly dissolved by the caustic and subcarbonated alkalies. With solutions of potash and soda, it forms pale brown compounds, from which, when recent, a little ammonia is evolved. In liquid ammonia the solution is of a reddish hue.

3. The action of the acids upon these different compounds is attended with nearly similar phenomena, a substance being separated intermediate in its properties between fat and albumen. Nitric acid, added in excess, re-dissolves this precipitate in the cold; and sulphuric, muriatic, and acetic, acids, when boiled upon it for a short time.

4. Neither alcohol nor ether exerts any action upon the coagulum of chyle; but, of the precipitate from its alkaline solution, they dissolve a small portion, which has the properties of spermacœti: the remainder is coagulated albumen.

5. Sulphuric acid very readily dissolves this coagulum, even when diluted with its weight of water; and, with the assistance of heat, it is soluble in a mixture of one part by weight of acid, with four of water; but, when the proportion of water is increased to six parts, the dilute acid exerts no action upon it. I was surprised to find that the alkalies produced no precipitation in these sulphuric solutions when heat had been employed in their formation, and where a small proportion only of the coagulum had been dissolved; and was therefore led to examine more particularly the changes which the coagulum had undergone by the action of the acid.

On evaporating a solution of one drachm of the coagulum in two ounces of dilute sulphuric acid (consisting of one part by weight of acid with three of water) down to one ounce, a small quantity of carbonaceous matter separated, and the solution had the following properties:

It was transparent, and of a pale brown color.

Neither the caustic nor carbonated alkalies produced in it any precipitation, when added to exact saturation of the acid, or in excess.

Infusion of galls, and other solutions containing tannin, rendered the acid solution turbid, and produced a more copious precipitation in that which had been neutralized by the addition of alkalies.

When evaporated to dryness, carbonaceous matter was deposited, and sulphurous acid evolved, with the other usual products of these decompositions.

6. On digesting the coagulum in dilute nitric acid, consisting of one part by weight of the acid to fifteen of water, it was speedily rendered of a deep brown color, but no other apparent change was produced for some weeks; when on removing it from the acid at the end of that period, it had acquired the properties of that modification of fat which is described by Fourcroy under the name of adepocire*.

A mixture of one part of nitric acid with three of water, acted more rapidly upon the coagulum of chyle; a portion of it was dissolved, and when the acid was carefully decanted from the remainder, it was found to possess the properties of gelatine. But, when heat was applied, or when a stronger acid was employed, the action became more violent, nitrogen and nitric oxide gas were evolved, and a portion of carbonic acid and of oxalic acid were produced.

7. Muriatic acid in its undiluted state does not dissolve the coagulum of chyle; but, when mixed with an equal quantity of water, or even more largely diluted, it dissolves it with facility, forming a straw-colored solution, which is rendered turbid when the alkalies are added to exact saturation; but no precipitate falls, nor can any be collected by filtration. When either acid or alkali is in excess in this solution, it remains transparent.

8. Acetic acid dissolves a small portion of the coagulum of chyle, when boiled upon it for some hours. As the solution cools, it deposits white flakes, which have the properties of coagulated albumen.

9. The action of oxalic acid is nearly similar to that of the acetic, but neither citric nor tartaric acid exert any action upon this coagulum.

---

* *Mem. de l'Acad. des Sciences*, 1789.

10. The

10. The destructive distillation of this substance affords water slightly impregnated with carbonate of ammonia, a small quantity of thin fetid oil and carbonic acid and carburetted hydrogen gas.

The coal which remains in the retort is of difficult incineration; it contains a considerable portion of muriate of soda and of phosphate of lime, and yields very slight traces of iron.

C. 1. The serous part of the chyle becomes slightly turbid when heated, and deposits flakes of albumen.

2. If, after the separation of this substance, the fluid be evaporated to half its original bulk, at a temperature not exceeding 200° Fahrenheit; small crystals separate on cooling, which, as far as I have been able to ascertain, bear a strong resemblance to sugar of milk: they require for solution about four parts of boiling water, and from sixteen to twenty parts of water of the temperature of 60°. They are sparingly soluble in boiling alcohol, but again deposited as the solution cools. At common temperatures alcohol exerts no action upon them. The taste of their aqueous solution is extremely sweet. By nitric acid they are converted into a white powder of very small solubility, and having the properties of saccholactic acid, as described by Scheele.*

The form of the crystals I could not accurately ascertain even with the help of considerable magnifiers. In one instance they appeared oblique six-sided prisms, but their terminations were indistinct.

Some of the crystals, heated upon a piece of platina in the flame of a spirit lamp, fused, exhaled an odor similar to that of sugar of milk, and burnt away without leaving the smallest perceptible residuum.

3. The destructive distillation of the serous part of chyle afforded a minute quantity of charcoal, with traces of phosphate of lime, and of muriate of soda, and carbonate of soda.

### Section III.—*Analysis of Lymph.*

The fluid found in the thoracic duct of animals that have been kept for twenty-four hours without food, is perfectly transparent and colorless, and seems to differ in no respect from that which is contained in the lymphatic vessels. It may therefore be regarded as pure lymph.

It has the following properties:†

1. It is miscible in every proportion with water.

* Chemical Essays, No. xvii.

† The term lymph has been applied indiscriminately to the tears, to the matter of encysted dropsy, and to some other animal fluids. Vide Aikin's Dictionary of Chemistry and Mineralogy, art. *Lymph.*

2. It produces no change in vegetable colors.

3. It is neither coagulated by heat, nor acids, nor alcohol; but is generally rendered slightly turbid by the last re-agent.

4. When evaporated to dryness, the residuum is very small in quantity, and slightly affects the color of violet paper, changing it to green.

5. By incineration in a platina crucible, the residuum is found to contain a minute portion of muriate of soda; but I could not discover in it the slightest indications of iron.

6. In the examination of this fluid, I availed myself with some advantage of those modes of electro-chemical analysis, which on a former occasion I have described to this Society.*

When the lymph was submitted to the electrical action of a battery consisting of twenty pairs of four-inch plates of copper and zinc, there was an evolution of alkaline matter at the negative surface, and portions of coagulated albumen were separated. As far as the small quantities on which I operated enabled me to ascertain, muriatic acid only was evolved at the positive surface.

### Section IV.—*Some Remarks on the Analysis of the Serum of Blood.*

This fluid has been so frequently and fully examined by chemists, that I shall not enter into a detailed account of its composition, but merely state such circumstances respecting it as relate particularly to the present inquiry, and have not hitherto been noticed by the experimentalists to whom I have alluded.

The fluid which oozes from serum that has been coagulated by heat, and which by physiologists is termed *serosity*, is usually regarded as consisting of gelatine, with some uncombined soda, and minute portions of saline substances, such as muriate of soda and of potash, and phosphate of lime, and of ammonia. Dr. Bostock regards it as mucus.†

From some experiments which I made upon the serum of blood, on a former occasion, I was induced to regard the serosity as a compound of albumen with excess of alkali, and to consider the coagulation of the serum analogous to that of the white of egg, and of the other varieties of liquid albumen.

To ascertain this point, and to discover whether gelatine exists in the serum, I instituted the following experiments.

Two fluid ounces of pure serum were heated in a water

* Phil. Trans. 1809, p. 373.

† Transactions of the Medical and Chirurgical Society of London, vol. i. p. 73.

bath bath until perfectly coagulated: the coagulum, cut into pieces, was digested for some hours in four fluid ounces of distilled water, which was afterwards separated by means of a filter.

The clear liquor reddened turmeric paper, and afforded a copious precipitation on the addition of infusion of galls, and, when evaporated to half an ounce, it gelatinized on cooling. It was rendered very slightly turbid by the addition of dilute sulphuric and muriatic acid; but alcohol produced no effect.

From the result of these trials, it might have been concluded that gelatine was taken up by the water; but, as an alkaline solution of albumen forms an imperfect jelly when duly concentrated, and as albumen and gelatine are both precipitated by tannin, I was inclined to put little reliance on the appearances just described, until I had examined the solution by the more accurate method of electrical decomposition.

Upon placing it in the Voltaic circuit, my suspicions were justified, by the rapid coagulation which took place in contact with the negative wire. I therefore made some other experiments in order to corroborate this result.

One fluid ounce of pure serum was dissolved in three of distilled water: the conductors from a battery of thirty pairs of four-inch plates were immersed in this solution at a distance of two inches from each other; the electrization was continued during three hours and a half, the solid albumen being occasionally removed: at the end of that period, no further coagulation took place, and a mere decomposition of the water was going on.

Having ascertained in previous researches, that gelatine is not altered during the electrical decomposition of its solution carried on as just described, my object in this experiment was, to ascertain whether any gelatine remained after the complete separation of the albumen had been effected. I accordingly examined the water from which the coagulated albumen had been removed, and found that it was not altered by infusion of galls, nor did it afford any gelatine when evaporated to dryness.

Two fluid ounces of dilute muriatic acid were added to one of serum. The mixture immediately assumed a gelatinous appearance; it was heated, and a more perfect coagulation of the albumen took place; the liquid part was separated by a filter. No effect was produced upon it by Voltaic electricity, nor did infusion of galls occasion any precipitation.

I repeated the first experiment with the addition of twenty drops drops of a solution of isinglass to the serum. The liquid which now separated, after the albumen had been entirely coagulated by the action of electricity, was copiously precipitated by infusion of galls.

It may be inferred from these experiments, that gelatine does not exist in the serum of the blood, and that the serosity consists of albumen in combination with a large proportion of alkali, which modifies the action of the re-agents commonly employed, but which is readily separated by electrical decomposition.

To ascertain whether iron exists in the serum of the blood, one pint was evaporated to dryness in a crucible, and gradually reduced to a coal, which was incinerated and digested in muriatic acid, to which a few drops of nitric acid were added; some particles of charcoal remained undissolved; the solution was saturated with ammonia, which afforded a copious precipitation of phosphate of lime, accompanied with slight traces only of oxide of iron.

Section V.—*Some Experiments upon the Coagulum of Blood.*

Mr. Hatchett's valuable researches on the chemical constitution of the varieties of coagulated albumen, have shown that the substance varies but little in its properties, whether obtained from the crassamentum of the blood, or from washed muscular fibre, or other sources; but that the proportion of earthy and saline matter is different in the different varieties.*

It will also be remarked, on referring to the dissertation which I have just quoted, that the ashes obtained by incinerating the coal left after the destructive distillation of albumen, did not contain any appreciable proportion of iron.

Assuming the existence of iron in the coloring matter of the blood, I made the following experiments upon the crassamentum of that fluid.

Two pints of blood were collected in separate vessels. The one portion was allowed to coagulate spontaneously; the other was stirred for half an hour with a piece of wood, so as to collect the coagulum, but to diffuse the principal part of the coloring matter through the serum. These two portions of coagulum were now dried in a water-bath, and equal weights of each reduced in a platina crucible to the state of coal, which afterwards was incinerated. The ashes were digested in dilute nitro-muriatic acid, and the solution saturated with liquid ammonia, in order to precipitate the

* Phil. Trans. 1800, p. 384.

phosphate of lime as well as any iron which might have been present.

The precipitates were collected, dried, and treated with dilute acetic acid, by which they were almost entirely dissolved, some very minute traces only of red oxide of iron remaining, the quantity of which was similar in both cases, and so small as nearly to have escaped observation.

It is reasonable to infer, if the coloring matter of the blood were constituted by iron in any state of combination, that a larger relative proportion of that metal would have been discoverable in the former than in the latter coagulum; but frequent repetitions of these experiments have shown that this is not the case, and the following result appears to complete the evidence on this subject.

The coloring matter of a pint of blood was diffused by agitation through the serum, from which it was allowed gradually to subside, the coagulum having been removed: after twenty-four hours, the clear serum was decanted off, and the remainder, containing the coloring matter, after having been evaporated to dryness, was incinerated, and the ash examined as in former experiments. But the traces of iron were here as indistinct as in the other instances above mentioned, although a considerable quantity of the coloring matter had been employed.

The minutiæ of analysis I have purposely excluded, as leading into details which would exceed the proper limits of this paper, and unnecessary in the present investigation. I shall now merely dwell on the principal results which have been obtained, and on the general conclusions which these afford.

Section VI.—*Researches on the coloring Matter of the Blood.*

1. To procure this substance for experiments, I generally employed venous blood which had been stirred during its coagulation; the fibrini is thus removed, and the coloring matter diffused through the serum, from which it gradually subsides, being difficultly soluble in that fluid; on decanting off the supernatant serum, the coloring matter remains in a very concentrated form. When other modes of procuring it were employed, they will be particularly mentioned; but, as I have not found the serum which is retained interfere much with the effects of various agents upon the coloring principle, the method just noticed was commonly adopted.

2. When the coloring matter thus collected is microscopically examined, it seems, as Lewenhoeck first observed,*

* Haller Elem. Physiolog. vol. i. p. 51.

to to consist of minute globules. These are usually described as soluble in water, a circumstance which my own observations led me to doubt, and which the more accurate experiments of Dr. Young, an account of which, intended for publication, he has kindly permitted me to peruse, have completely disproved.

3. The effect of water upon the red globules, is to dissolve their coloring matter, the globule itself remaining colorless, and, according to Dr. Young, floating upon the surface.

This aqueous solution is of a bright red color, and not very prone to putrefaction. When heated, it remains unaltered at temperatures below 190° or 200° Fahrenheit; at higher temperatures it becomes turbid, and deposits a pale brown sediment: if in this state it be poured upon a filter, the water passes through without color, so that exposure to heat not only destroys the red tint, but renders the coloring matter insoluble in water.

Alcohol and sulphuric ether added to this solution also render it turbid; and, when these mixtures were filtrated, a colorless and transparent liquor was obtained.

4. The matter remaining upon the filter was insoluble in water, in alcohol, and in sulphuric ether; but, when digested in dilute muriatic or sulphutic acid, a portion was taken up forming a brown solution. I regard this soluble portion as a modification of the coloring matter produced by the operation of heat: the insoluble residuum had the properties of albumen.

5. *Effects of Acids on the coloring Matter.*—A. Muriatic acid poured upon the coloring matter of the blood, renders one portion of it nearly insoluble, and of a bright brown color: another portion is taken up by the acid forming a dark crimson solution when viewed by reflected light; but, when examined by transmitted light, it has a greenish hue.

This solution remains transparent, and its color is unimpaired by long exposure to light, either in contact with the air, or when kept in close vessels. At its boiling temperature the color is also permanent.

Infusion of galls produces no change in this muriatic solution, nor is its color affected by carbonated alkalies, even when added in considerable excess.

It is rendered brown-red by supersaturation with caustic potash, but not with soda, nor ammonia: these, and especially the latter, rather heighten its color.

When considerably diluted with water, its original color is much impaired, and the green hue, which it always exhibits by transmitted light, becomes more evident.

In preparing this solution, I frequently employed the

 coagulum coagulum of blood cut into pieces, and digested in equal parts of muriatic acid and water, at a temperature between 150° and 200°. In three or four hours the acid was poured off, and filtrated. The clear solution was in all respects similar to that above described, although before filtration it appears of a dirty brown color.

I evaporated a portion of this muriatic solution in a water-bath, to dryness; it retained its color to the last, and left a transparent pellicle upon the evaporating bason, of a dirty red color: this, when redissolved in muriatic acid, acquired its former tint, but the color of its aqueous solution was nearer brown than red.

B. Sulphuric acid, diluted with eight or ten parts of water, forms an excellent solvent of the coloring principle of the blood.

It may be employed in a more concentrated state; but the bright color of the solution is in that case apt to be impaired, and, when more largely diluted with water, its action is slow and uncertain. Either the sediment of the coloring matter from the serum, or the crassamentum of the blood, may be indifferently employed in forming these solutions.

When dilute sulphuric acid is added to the coloring matter, it renders it slightly purple; and if no heat be applied, the acid when poured off and filtered, is colorless; so that dilute sulphuric acid when cold, does not dissolve this coloring principle.

One part of the crassamentum of blood cut into pieces, was put into a matrass placed in a sand heat, with about three parts of dilute sulphuric acid. It was kept for twelve hours in a temperature never exceeding 212°, nor below 100°. After twenty-four hours the acid was filtered off, and it exhibited a beautiful bright lilac color, not very intense, and teinted with green when viewed by transmitted light.

This solution is nearly as permanent as that in the muriatic acid. Some of it, which has been kept for a month in an open vessel, often exposed to the direct rays of the sun, is very little altered.

When diluted with two or three times its bulk of water, the lilac tint disappears, and the mixture is only slightly green.

When exposed to heat, the color gradually changes as the acid becomes more concentrated by evaporation, and when reduced to about half its bulk the lilac hue is destroyed.

The solutions of pure and carbonated alkalies when added in in excess, convert the color of this sulphuric solution to brownish red; but, in smaller quantities, they merely impair it by dilution.

C. Nitric acid, even much diluted, is inimical to the coloring matter of the blood.

A few drops added to the muriatic or sulphuric solutions gradually convert their color to a bright brown, and larger quantities produce the same change immediately.

The action which this acid exerts upon the coloring matter under other circumstances is nearly similar, and always attended with its decomposition; so that my attempts to procure a red solution in this menstruum uniformly failed of success.

D. Acetic acid dissolves a considerable quantity of the coloring matter of the blood; the solution is of a deep cherry-red color. When somewhat diluted, or when observed in tubes of about a quarter of an inch bore, this solution appears perfectly green by transmitted light. In its other habitudes it nearly resembles the muriatic solution.

E. The solution of the coloring matter in oxalic acid is of a brighter red than those hitherto noticed; that in citric acid is very similar to the acetic solution, and with tartaric acid the compound somewhat inclines to scarlet. All these solutions exhibit the green hue, to which I have so often alluded, in a remarkable degree.

6. *Effects of Alkalies on the coloring Principle of the Blood.*—The caustic and the carbonated alkalies form deep red solutions of this substance, which are extremely permanent.

1. Solutions of pure potash, and of the subcarbonate, take up a large proportion of the coloring matter of the blood. The intensity of the color of this solution, when concentrated, is such, that it appears opake, unless viewed in small masses, or in a diluted state, when it is of a bright red color.

2. In soda and its subcarbonate, the solution has more of a crimson hue, which color is extremely bright in its concentrated state.

3. The solution in liquid ammonia approaches nearer to scarlet than that in which the fixed alkalies are employed.

4. When these alkaline solutions are supersaturated with muriatic acid, or with dilute sulphuric acid, they acquire a color nearly similar to the original solutions in those acids which have been above described.

5. Nitric acid added in small quantities, or even to saturation of the alkaline menstruum, heightens the color of the three compounds; but, when there is a slight excess, a tint tint of orange is produced, which soon passes into bright yellow.

6. The alkaline solutions may be evaporated nearly to dryness without losing their red colors; during the evaporation of the ammoniacal solution, the alkali flies off, and a brown-red solution of the coloring matter in water remains.

Having ascertained the above facts respecting the coloring principle of the blood, I next proceeded to examine how far it was susceptible of entering into those combinations which are peculiar to other varieties of coloring matter.

These experiments I shall detail in the order in which they were made.

1. Some pure alumine was added to a concentrated aqueous solution of the coloring matter of the blood, and after twenty-four hours the mixture, which had been frequently agitated during that period, was poured upon a filter, and the residuum washed with hot distilled water.

The filtrated liquor had lost much of its original color; the alumine had acquired a red tinge; it was dried at a temperature between 70° and 80°, during which it became brown.

2. Two hundred grains of alum were dissolved in four fluid ounces of a solution of the coloring matter, similar to that employed in the last experiment. The color of the compound was bright red. Liquid ammonia was added, and the precipitate collected, and carefully dried. It was of a dirty red, and after some days' exposure to light became nearly brown.

From these, and other experiments which I have not thought it necessary to detail, it appears that alumine will not form a permanent red compound with the coloring principle of the blood; I was therefore next induced to employ oxide of tin.

3. Fifty grains of crystallized muriate of tin (prepared by boiling tin filings in muriatic acid, and evaporating the solution,) were dissolved in four ounces of the solution of coloring matter, which immediately assumed a purple tint, and became afterwards brown. It was diluted with twice its bulk of water, and put aside in a stopped phial. On examining it three days afterwards, a small quantity of a bright red powder was observed at the bottom of the phial, which proved to consist of the coloring principle combined with the metallic oxide. A portion of this compound which has been kept in water for some weeks has undergone no change of color; but, when dried by exposure to air, it loses its brilliant tint, and becomes of a dull red hue.

To

To a compound solution of muriate of tin and coloring matter, similar to that employed in the last experiment, I added a sufficient quantity of solution of potash to decompose the salt of tin. The precipitate thus obtained was collected, and dried by exposure to the air of a warm room. It was of a dull red color, and has undergone no apparent change by exposure to the joint action of light and air for three weeks.

4. Finding that supertartrate of potash exalted the color of the blood, I endeavoured to form a compound of it with that substance and oxide of tin, and thus, in some measure, to imitate the process in which cochineal is employed for the production of scarlet dye; but, although a bright red compound is produced, when it is dried at a very moderate temperature its color becomes similar to that of the other combinations which I have described.

These experiments I repeated in various ways, occasionally applying the salt of tin as a mordant to woollen cloth, linen, &c.; but the brilliancy of the color was never permanent.

5. Having observed that infusion of galls and decoction of oak bark do not impair the color of the blood, I conceived that solution of tannin might answer the purpose of a mordant, as it is effectually employed by dyers in giving permanence to some of their red colors.

I accordingly impregnated a piece of calico with decoction of oak bark, and afterwards passed it through an aqueous solution of the coloring matter of blood. When dry, it was of a dirty red color, nearly similar to that which would have been obtained had no mordant been applied: when, however, an alkaline solution of the coloring matter was employed, the color was equal to that of a common madder red, and, as far as I have been able to ascertain, it is permanent.

6. A solution of superacetite of lead was impregnated with the coloring matter of the blood. The compound was bright red: no spontaneous change took place in it, and on the addition of an alkali a white precipitate was formed, the fluid retaining its former tint.

From this and other experiments, in which it was attempted to combine oxide of lead with the coloring of the blood, it would appear that there is no attraction between those two substances.

7. The most effectual mordants which I have discovered for this coloring matter, are some of the solutions of mercury, especially the nitrate, and corrosive sublimate.

Ten grains of nitrate of mercury (prepared with heat and containing the red oxide) were dissolved in two fluid ounces of of a solution of the coloring of the blood. After some hours a deep red compound was deposited, consisting chiefly of the metallic oxide combined with the coloring matter, and a small portion of coagulated albumen. The remaining fluid had nearly lost its red color.

The nitrate of mercury containing the black oxide, produces nearly similar effects, excepting that the color of the compound is of a lighter red.

When corrosive sublimate is added to the solution of the coloring matter, its tint is instantaneously brightened, and it becomes slightly turbid from the deposition of albumen. If this be immediately separated by a filter, the liquor which passes through gradually deposits a deep red or purplish insoluble precipitate, and if it now be again filtrated the liquor is colorless, the whole of the coloring principle being retained in the compound which remains upon the filter.

By impregnating some pieces of woollen cloth with solution of nitrate of mercury, or of corrosive sublimate, and afterwards steeping then in an aqueous solution of the coloring matter of the blood, I succeeded in giving them a permanent red tinge, unalterable by washing with soap; and, by employing the ammoniacal solution of the coloring matter, calico and linen may be dyed with the same mordant.

In these experiments I was much satisfied by the complete separation of the coloring matter from its solutions, which after the process were perfectly colorless.

### Section VII.—*Some Remarks on the preceding experimental Details.*

From the experiments related in the second section of this paper, it appears that sulphuric acid effects changes upon the coagulum of chyle, similar to those which Mr. Hatchett has observed to result from the action of dilute nitric acid upon the coagulated white of egg. This last substance, however, is not convertible into gelatine by means of sulphuric acid; whereas in these respects the curd of milk resembles that of chyle: this circumstance, as well as the more ready solubility of the coagulum of chyle in dilute than in concentrated acids, points out a strong analogy between those two bodies.

The sweet taste of chyle naturally suggested the idea of its containing sugar;* but I am not aware of any direct experiments which have demonstrated its existence, and have therefore detailed minutely such researches as I have been

* Fordyce on Digestion, 2d edit. p. 121.

enabled enabled to make upon the subject, hoping at some future period to render them more complete.

The experiments to prove the non-existence of gelatine in the serum of blood, will, I trust, be deemed sufficiently decisive: they show that that abundant proximate principle of animals is not merely separated from the blood, in which it has been supposed to exist ready formed, but that it is an actual product of secretion.

The proportion of iron afforded by the incineration of several varieties of animal coal, is much less considerable than we have been led to expect; and the experiments noticed in the fifth section show that it is not more abundant in the coloring matter of the blood than in the other substances which were submitted to examination; and that traces of it may be discovered in the chyle which is white, in the serum, and in the washed crassamentum or pure fibrina.

The inferences to which I have alluded, in the first section of this paper, are strongly sanctioned by these facts, and coincide with the opinion which has been laid before the Royal Society, by Dr. Wells,* respecting the *peculiar nature* of the coloring principle of the blood, and support the arguments which are there adduced.

That the coloring matter of the blood is perfectly independent of iron, is, I conceive, sufficiently evident from its general chemical habitudes, and it appears probable that it may prove more useful in the art of dyeing than has hitherto been imagined, since neither the alkalies nor the acids (with the exception of the nitric) have much tendency to alter its hue. The readiness, too, with which its stains are removed from substances to which no mordant has been applied, seem to render it peculiarly fit for the purposes of the calico-printer. I have not extended these experiments, nor have I had them repeated on a sufficient scale to enable me to draw more general conclusions respecting the possibility of applying them with advantage in the arts: this would have led me into too wide a field, and one not immediately connected with the objects of this Society: the subject, however, appears important.

It is not a little remarkable that blood is used by the Armenian dyers, together with madder, in the preparations of their finest and most durable reds†, and that it has even been found a necessary addition to insure the permanency

* Phil. Trans. 1797.

† Tooke's Russian Empire, vol. iii. p. 497.

of the color.* This fact alone may be regarded as demonstrating the non-existence of iron as the coloring principle of the blood, for the compounds of that metal convert the red madder to grey and black.

Whilst engaged in examining the coloring matter of the blood, I received from Mr. William Money, house-surgeon to the general hospital at Northampton, some menstruous discharge, collected from a woman with prolapsus uteri, and consequently perfectly free from admixture of other secretions. It had the properties of a very concentrated solution of the coloring matter of the blood in a diluted serum, and afforded an excellent opportunity of corroborating the facts respecting this principle, which have been detailed in the preceding pages. Although I could detect no traces of iron, by the usual modes of analysis, minute portions of that metal may and probably do exist in it, as well as in the other animal fluids which I have examined; but the abundance of coloring matter in this secretion should have afforded a proportionable quantity of iron, did any connection exist between them. It has been observed that the artificial solutions of the coloring matter of the blood invariably exhibit a green tint when viewed by transmitted light: this peculiarity is remarkably distinct in the menstruous discharge.†

I hope that some of the facts furnished by the above experiments may prove useful to the physiological inquirer: they account for the rapid re-production of perfect blood after very copious bleedings, which is quite inexplicable upon that hypothesis which regards iron as the coloring matter, and may perhaps lead to the solution of some hitherto unexplained phenomena connected with the function of respiration. There can, I think, be little doubt that the formation of the coloring matter of the blood is connected with the removal of a portion of carbon and hydrogen from that fluid, and that its various tints are dependent upon such modifications of animal matter, and not, as some have assumed, upon the different states of oxidizement of the iron which it has been supposed to contain.—*Phil. Trans.*

---

* Aikin's Dictionary, art. *Dyeing*, and Philos. Mag. vol. xviii.

† I could discover no globules in this fluid; and, although a very slight degree of putrefaction had commenced in it, yet the globules observed in the blood would not have been destroyed by so trifling a change.

CRITICAL

